# Physical–chemical properties of cell wall interface significantly correlated to the complex recalcitrance of corn straw

**DOI:** 10.1186/s13068-021-02047-0

**Published:** 2021-10-01

**Authors:** Yufen Wang, Xianyang Xu, Huiting Xue, Dejian Zhang, Guanhua Li

**Affiliations:** 1 Key Laboratory of Herbage and Endemic Crop Biotechnology, School of Life Sciences, Inner Mongolia University, 49 Xilin Road, Hohhot, 010070 China; 2 State Key Laboratory of Reproductive Regulation and Breeding of Grassland Livestock, School of Life Sciences, Inner Mongolia University, 49 Xilin Road, Hohhot, 010070 China; 3 College of Basic Medicine, Inner Mongolia Medical University, Jinshan Road, Hohhot, 010110 China

**Keywords:** Recalcitrant barrier, Pore, Enzymatic hydrolysis, Cell wall, Corn straw

## Abstract

**Background:**

Tissue heterogeneity significantly influences the overall saccharification efficiency of plant biomass. However, the mechanisms of specific organ or tissue recalcitrance to enzymatic deconstruction are generally complicated and unclear. A multidimensional analysis of the anatomical fraction from 12 corn cultivars was conducted to understand the essence of recalcitrance.

**Results:**

The results showed that leaf, leaf sheath, stem pith and stem rind of corn straw exhibited remarkable heterogeneity in chemical composition, physical structure and cell type, which resulted in the different saccharification ratio of cellulose. The high saccharification ratio ranging from 21.47 to 38.96% was in stem pith, whereas the low saccharification ratio ranging from 17.1 to 27.43% was in leaf sheath. High values of lignin, hemicelluloses, degree of polymerization and crystallinity index were critical for the increased recalcitrance, while high value of neutral detergent soluble and pore size generated weak recalcitrance. Interestingly, pore traits of cell wall, especial for microcosmic interface structure, seemed to be a crucial factor that correlated to cellulase adsorption and further affected saccharification.

**Conclusions:**

Highly heterogeneity in cell wall traits influenced the overall saccharification efficiency of biomass. Furthermore, the holistic outlook of cell wall interface was indispensable to understand the recalcitrance and promote the biomass conversion.

**Graphic abstract:**

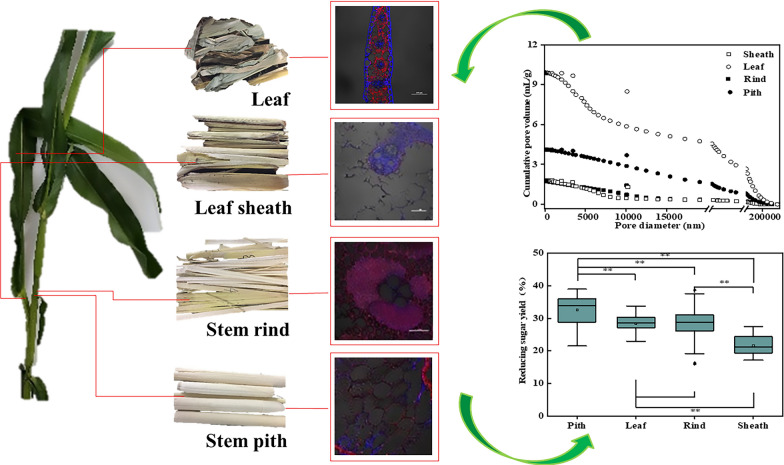

**Supplementary Information:**

The online version contains supplementary material available at 10.1186/s13068-021-02047-0.

## Background

Governments and energy corporations around the world are actively boosting the efficient utilization of abundant, renewable and bio-degradable feedstocks, due to the exhaustion of fossil resources and the severe environmental pollution [1]. The output of lignocellulosic biomass can reach 181.5 billion tonnes annually [2]. Lignocellulosic biomass refers to the biological feedstocks, mainly including agricultural products, forestry wastes, and industrial residues. These feedstocks can be potentially converted to bioenergy, biochemicals and other bioproducts based on the biorefinery [3]. An integrated biorefinery is a sustainable facility involving in various physical, thermochemical and biochemical approaches, etc. [4]. It is largely acknowledged that saccharification of cellulose with economic competitiveness is deemed as one of the core requirements in biomass-based biorefinery.

Enzyme-catalyzed saccharification of cellulose is the most optimal approach, yet, the efficiency in this process is commonly hampered by various factors. Series of physical and chemical factors that hinder the enzymatic saccharification of cellulose are deemed as “recalcitrance”, which is referred as (but are not limited to) composition (consisting of cellulose, lignin, hemicelluloses and pectin), polymer properties (such as lignin monomer components, cellulose crystalline index and degree of polymerization), and interactive network among these polymers [5]. Assessing and destroying the recalcitrance are prerequisite to circumvent the technical and economic obstacle in recent circular bioeconomy.

Plant biomass has evolved various functional and morphological fractions involving in complicated constituents and structures, which is named as ‘‘heterogeneity”. In terms of hemicelluloses and lignin, the amount, distribution and their decoration pattern add complexity to the origins and tissues of plant biomass [6]. Crowe also indicates lignin content commonly exhibits a negative correlation with saccharification ratios, yet, is not the sole contributor to recalcitrance of switchgrass [7]. Thus, a consensus can be reached that the high heterogeneity of plant biomass leads to the significant complexity of recalcitrance, which further influences the overall conversion efficiency of plant biomass. A further complication is that the complexity commonly causes empirically implemented enzymatic saccharification process and drastic pretreatment for recalcitrance more than necessary [8]. Hence, this immense heterogeneity is considered as the chief reason why the precise mechanisms of recalcitrance are still ambiguous and also closely influence the optimal choice of pretreatment and enzymatic hydrolysis strategy. Additionally, it is seemingly difficult to systematically compare these qualities to evaluate the intrinsic recalcitrance among different feedstocks due to the heterogeneity, especially for different studies.

Dried plant biomass is primarily composed of cell wall. The essence of enzymatic saccharification is the hydrolysis of cellulose in cell wall [9]. The enzymatic saccharification correlates to the properties of cell wall including the relative abundances of composition, fine proportion, their interconnecting among different matrices and multiple structures [10]. The basic types of cells among various plants are overall similar. Varied behaviors of different cell types in enzymatic hydrolysis reflect the tissue‑specific recalcitrance that influenced by the nature of cell wall [11, 12]. Thus, comprehending the resistant factors with viewpoint of cell wall might provide a deep insight into the recalcitrance of plant biomass. Allowing the enzyme access to the cell wall is the primary step in enzymatic hydrolysis of cellulose, which significantly influences the latter saccharification process [13]. However, the susceptibility of different cell wall, especially the accessibility of bio-interface to enzyme is still unclear.

Corn is the main agricultural crop on the earth. Corn straw (CS) is the main residue of corn. Based on the desirable agronomic and biochemical properties in terms of high biomass yield and cellulose content, and C4 carbon fixation, CS has attracted huge attention among researchers for biomass biorefinery. From visual angle, it is easily found that leaf, leaf sheath, stem rind and stem pith have different morphologies. Thus, CS is especially suitable model to clarify tissue-specific recalcitrance for its greater diversity of cells than other plant biomass. Although enzymatic saccharification of cellulose from CS has been studied by many researchers, little investigation concerning the relevance of enzymatic saccharification to heterogeneity in composition and architecture of specific organs or tissues has been reported. In an effort to clarify the fundamental knowledge about origins of recalcitrance, enzymatic saccharification and physical–chemical properties of cell wall in corn cultivars with different macroscopic phenotypes were determined. And the relationship between multidimensional properties of cell wall and the enzymatic digestibility of cellulose were discussed. This paper focused on understanding the recalcitrance of cell wall interface, which will also help in selecting of corn varieties with improved biorefining capabilities and setting downstream pretreatment.

## Results and discussion

### Saccharification performance

**Fig. 1:**
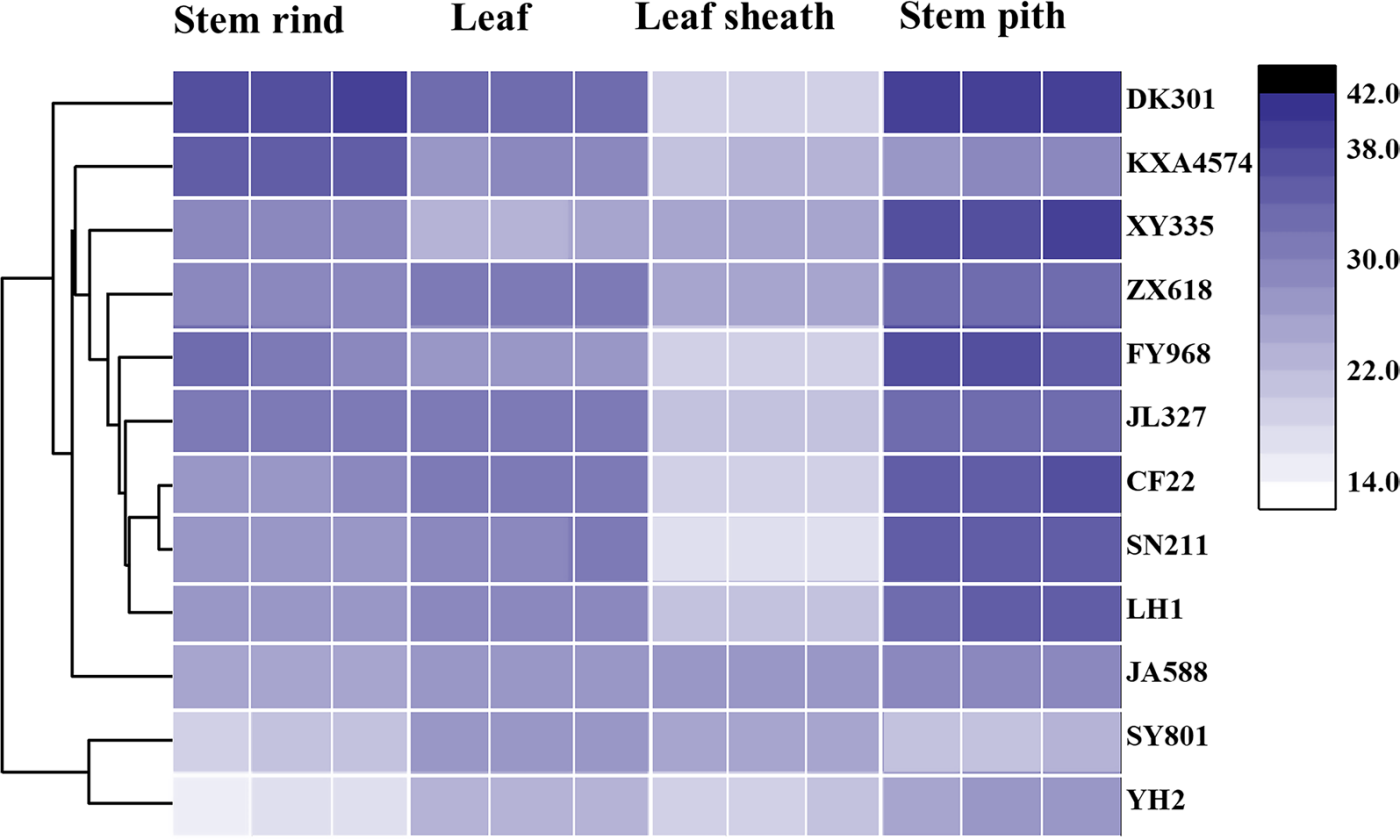
The cluster heatmap of saccharification ratios of different fractions from 12 corn cultivars. The selected fractions were leaf, leaf sheath, stem rind and stem pith, respectively. The color legend of dark purple to light purple representing saccharification ratios were automatically generated at the top right side of the figure

**Fig. 2:**
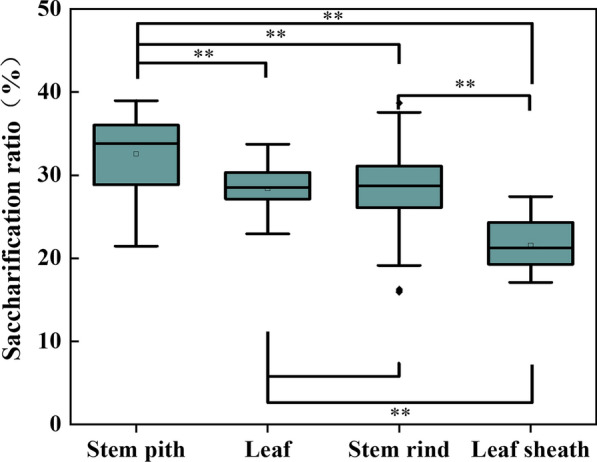
Saccharification ratios of different fractions from 12 corn cultivars. One-way ANOVA were carried out among various fractions. **Represented *P* < 0.01

The 144 saccharification ratio values of the four anatomical fractions from 12 corn cultivars were used to generate a heat map contained in a matrix with legend color bar. Dendrograms were selected to describe the similarity between clusters. Hierarchical clustering analysis confirmed the initial idea that some cultivars necessarily had a comparable performance in saccharification (Fig. 1). Additionally, there were no obvious correlations between the saccharification ratio and the macroscopic phenotypes of corn straw such as height, dry weight, etc. The general varying patterns for saccharification ratio among the same fraction within the different cultivar was similar, suggesting that biomass saccharification obviously correlated to the specific organs or tissues (Fig. 2). The highest saccharification ratio was obtained from stem pith, and followed by leaf, stem rind and leaf sheath in sequence, despite the difference between the stem pith and leaf was not significant. Specially, the saccharification ratio of stem pith ranged from 21.47 to 38.96%, whereas it ranged from 17.1 to 27.43% among leaf sheath.

Enzymatic digestibility expressed as saccharification ratio can be used to reflect the recalcitrance. Higher enzymatic digestibility indicates weaker recalcitrance [14]. The outermost leaf sheath was the most recalcitrant fraction, and followed by the stem rind. The leaf was the least recalcitrant fraction except for the stem pith. Hence, recalcitrance exhibited obvious heterogeneity among organs or tissues. Li also reports that different fractions of sorghum with structural or chemical variations generate special recalcitrance [15]. This lack of agreement about recalcitrance of leaf, leaf sheath, stem rind and stem pith indicated the complication of cell wall assembly, which might further translate dictate recalcitrance of cell wall to sugar production. Saccharification of cellulose is an enzymatic hydrolysis of cell wall in plant, exhibiting as glucose release from cellulose. Thus, the relationships between the enzymatic digestibility and physiochemical characteristics of cell wall were analyzed.

### Associations between cell wall properties and their impact on enzymatic digestibility

Representative DK and YH were chosen to the further study, because they were expected to exhibit extreme and contrasting phenotypes. DK had the highest biomass and height among the 12 cultivars, yet, YH represented the opposite phenotypes (the lowest biomass and height). Physical–chemical properties were determined for each leaf, leaf sheath, stem rind and stem pith, and used to evaluate the relationship between cell wall and recalcitrance. The values of enzymatic digestibility were different for the stem pith and stem rind across DK and YH, yet similar for the leaf sheath (Additional file 3: Table S2). Overall, enzymatic digestibility of leaf, stem rind and stem pith were significantly higher in DK than in YH (*P* < 0.01). Detailed information concerning the different physical–chemical properties and significance analysis is provided in Tables 1 and 2. The general varying patterns for physical–chemical properties within the same anatomical fraction were similar. The crystallinity index, degree of polymerization, and ash values were higher in stem rind and leaf sheath than in stem pith and leaf. Leaf sheath showed the least content of NDS, whereas leaf contained the highest content of NDS, due to the large amounts of soluble sugars and protein. Similar trend is also observed in sorghum stem [16]. Higher crystallite dimension and DB/DO ratios were in stem pith or leaf. Moreover, higher cellulose, hemicelluloses and lignin values were in stem rind or leaf sheath. The collective data confirmed that characteristics of cell wall from different organs or tissues were related to the varied recalcitrance.

**Table 1:** Physical–chemical properties for four anatomical fractions of various DK 301 samples

Fraction	Physical properties				Chemical properties				
	CrI	DP	CD (nm)	DO/DB	Cel (%)	NDS (%)	H-Cel (%)	Lig (%)	Ash (%)
Leaf	23.89 ± 0.92^a^	474.42 ± 10.74^a^	17.70 ± 1.70^a^	0.84 ± 0.015^a^	39.76 ± 1.08^a^	21.70 ± 1.20^a^	27.20 ± 1.56^a^	7.99 ± 0.12^a^	3.23 ± 0.12^a^
Leaf sheath	36.18 ± 0.051^b^	746.39 ± 0.46^b^	14.19 ± 0.04^b^	0.74 ± 0.01^b^	35.08 ± 0.31^b^	4.68 ± 0. 50^b^	41.42 ± 0.50^b^	16.23 ± 0.478^b^	2.25 ± 0.090^b^
Stem rind	42.06 ± 0.97^c^	761.75 ± 10.22^b^	10.10 ± 0.80^c^	1.29 ± 0.02^c^	45.65 ± 0.31^c^	15.28 ± 0.52^c^	20.77 ± 1.26^c^	13.88 ± 0.39^c^	4.18 ± 0.066^c^
Stem pith	23.57 ± 0.27^a^	351.76 ± 2.66^c^	18.65 ± 0.65^a^	1.73 ± 0.01^d^	40.79 ± 0.04^a^	14.71 ± 1.17^c^	33.51 ± 1.10^d^	7.54 ± 0.11^a^	2.52 ± 0.12^d^

The data labeled by the different superscripts (a–d) within the same column were different from each other (*P* < 0.05)

**Table 2:** Physical–chemical properties for four anatomical fractions of various YH 2 samples

Fraction	Physical properties				Chemical properties				
	CrI	DP	CD (nm)	DO/DB	Cel (%)	NDS (%)	H-Cel (%)	Lig (%)	Ash (%)
Leaf	44.35 ± 1.68^a^	726.82 ± 2.89^a^	19.80 ± 0.40^a^	1.30 ± 0.0058^a^	26.14 ± 0.81^a^	19.80 ± 1.05^a^	31.74 ± 0.74^a^	20.59 ± 1.93^a^	1.69 ± 0.24^a^
Leaf sheath	35.54 ± 0.13^b^	706.02 ± 0.49^b^	13.85 ± 0.35^b^	0.73 ± 0.0058^b^	36.81 ± 0.15^b^	5.28 ± 0.57^b^	37.36 ± 1.03^b^	15.19 ± 0.76^b^	5.28 ± 0.049^b^
Stem rind	55.39 ± 0.12^c^	1124.75 ± 2.66^c^	10.15 ± 0.35^c^	0.83 ± 0.01^c^	31.57 ± 1.18^c^	11.64 ± 0.085^c^	23.79 ± 0.59^c^	26.44 ± 1.82^c^	6.27 ± 0.035^c^
Stem pith	33.96 ± 1.76^b^	457.01 ± 3.90^d^	13.50 ± 0.50^b^	1.70 ± 0.01^d^	29.73 ± 1.16^d^	16.53 ± 1.43^d^	29.02 ± 1.99^d^	21.65 ± 0.62^a^	2.27 ± 0.031^d^

The data labeled by the different superscripts (a–d) within the same column were different from each other (*P* < 0.05)

**Fig. 3:**
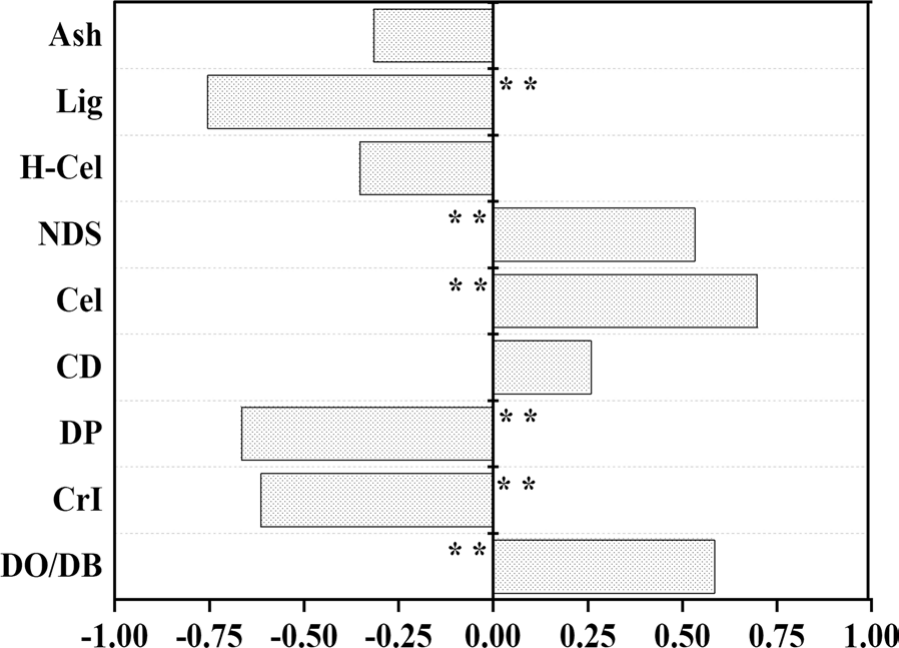
Correlation analysis between biomass properties and enzymatic digestibility for DK 301 and YH 2

A correlation matrix was performed to evaluate how the recalcitrance was associated with cell wall properties (Fig. 3). The results showed that the contents of cellulose and NDS positively impacted the enzymatic digestibility. The positive effect of cellulose is not surprising for it probably reflected a greater availability of substrate for enzymatic hydrolysis. Although NDS might interact with hemicellulose and lignin to influence the enzymatic digestibility of cellulose, higher content of NDS commonly indicated the lower relative content of other relative recalcitrance [17]. The influences of ash and crystallite dimension among the subset of samples on enzymatic digestibility were not statistically significant. The negative impact of lignin, degree of polymerization and crystallinity index values on enzymatic hydrolysis were in accordance with the recalcitrance model reported in the previous literature [18, 19]. The negative impact of hemicelluloses was not statistically significant. Because, the physical barriers of hemicelluloses to block cellulose is highly correlated to the ultrastructure of cellulose especially for the accessibility of cellulose surface [20]. Additionally, the leaf sheath had intermediate lignin content and crystallinity index compared to the other tissues yet the lowest DO/DB ratio and digestibility, suggesting the DO/DB ratio had a positive effect on enzymatic digestibility. The DO/DB ratio determined by Simon’s staining can be used to describe the relative interior and exterior accessibility of pore [21]. Enzyme accesses to the cellulose core needs to be in intimate and prolonged contact [22]. Pore properties involving in volume, specific surface, tortuosity, size and fractal dimension might all affect accessibility of cellulose. Thus, pore characteristics and size distribution of various samples were determined.

### Desirable pore traits of cell wall for enzymatic digestibility

**Fig. 4:**
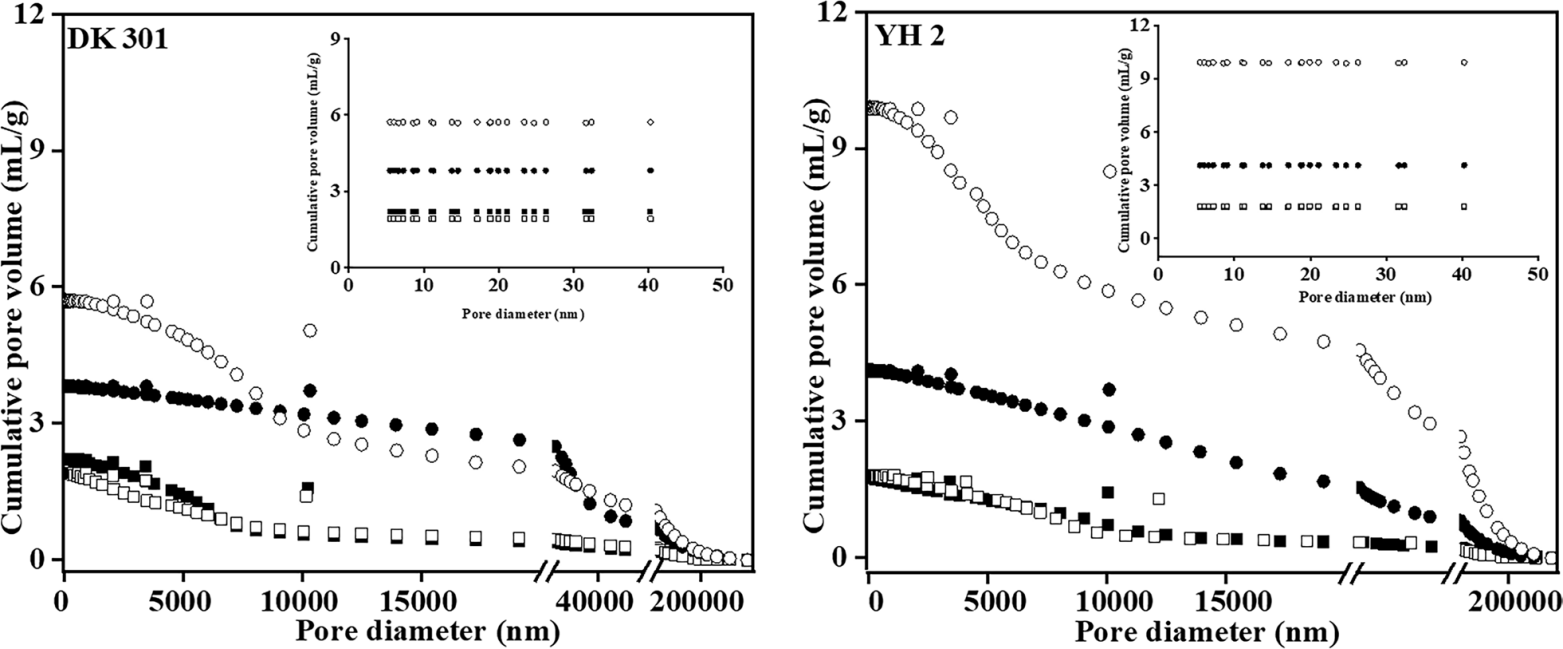
Pore size distribution of different fractions for DK301 and YH 2. Black square represented stem rind. White square represented leaf sheath. Black dot represented leaf. White dot represented stem pith

Generally, the leaf had the highest average pore diameter, whereas the stem pith had the highest total pore area and porosity (Table 3). These results were significantly positively correlated with enzymatic digestibility, indicating that higher pore size and more pore number could facilitate enzymatic hydrolysis. Differential intrusion volume vs. diameter was also plotted (Fig. 4). The pore size distribution trends of DK and YH were similar. The different pore especially for the class corresponding to the pore size ranging from 5 to 15 nm obviously correlated to the enzymatic digestibility. Plant biomass can be deemed as special porous material, consisting of highly ordered porous structure at various levels, such as cell lumen, inter-cellular spaces, pit in cell wall, and space among chemical polymers. Arantes suggests that pore size distribution is mainly responsible for the efficiency of enzymatic hydrolysis [23]. Sun suggests that the specific structure of pore not the pore volume impacts the efficiency of enzymatic hydrolysis [24]. A large fraction of the pore volume might be ascribed to pores with small size. Pores with size bigger than 5.1 nm are sufficient for accessibility of cellulase, but small pores might resist mass transfer, thus hindering enzymatic hydrolysis [25]. In sum, catalytic cleavage of the cellulose was not the actual rate-limiting step, but rather the limited accessibility of the cellulose chains to the enzyme within the substrate. Pore size instead of volume could accurately represent the relationship between the porous structure and accessibility of the cellulose.

**Table 3:** Porous parameters of four anatomical fractions for DK 301 and YH 2

Cultivar	Fraction	Total volume (mL/g)	Total pore area (m^2^/g)	Average pore diameter (nm):	Porosity (%)
DK 301	Leaf	3.82	1.53	10,002.15	84.18
	Leaf sheath	1.92	2.94	2614.89	71.33
	Stem rind	2.20	2.65	3330.04	77.40
	Stem pith	5.72	3.35	6825.19	88.25
YH 1	Leaf	4.13	2.36	7009.25	84.85
	Leaf sheath	1.83	2.43	3010.86	72.88
	Stem rind	1.75	2.22	3140.22	71.30
	Stem pith	9.91	6.33	6263.86	90.00

### Morphology variation and cellulase adsorption

**Fig. 5:**
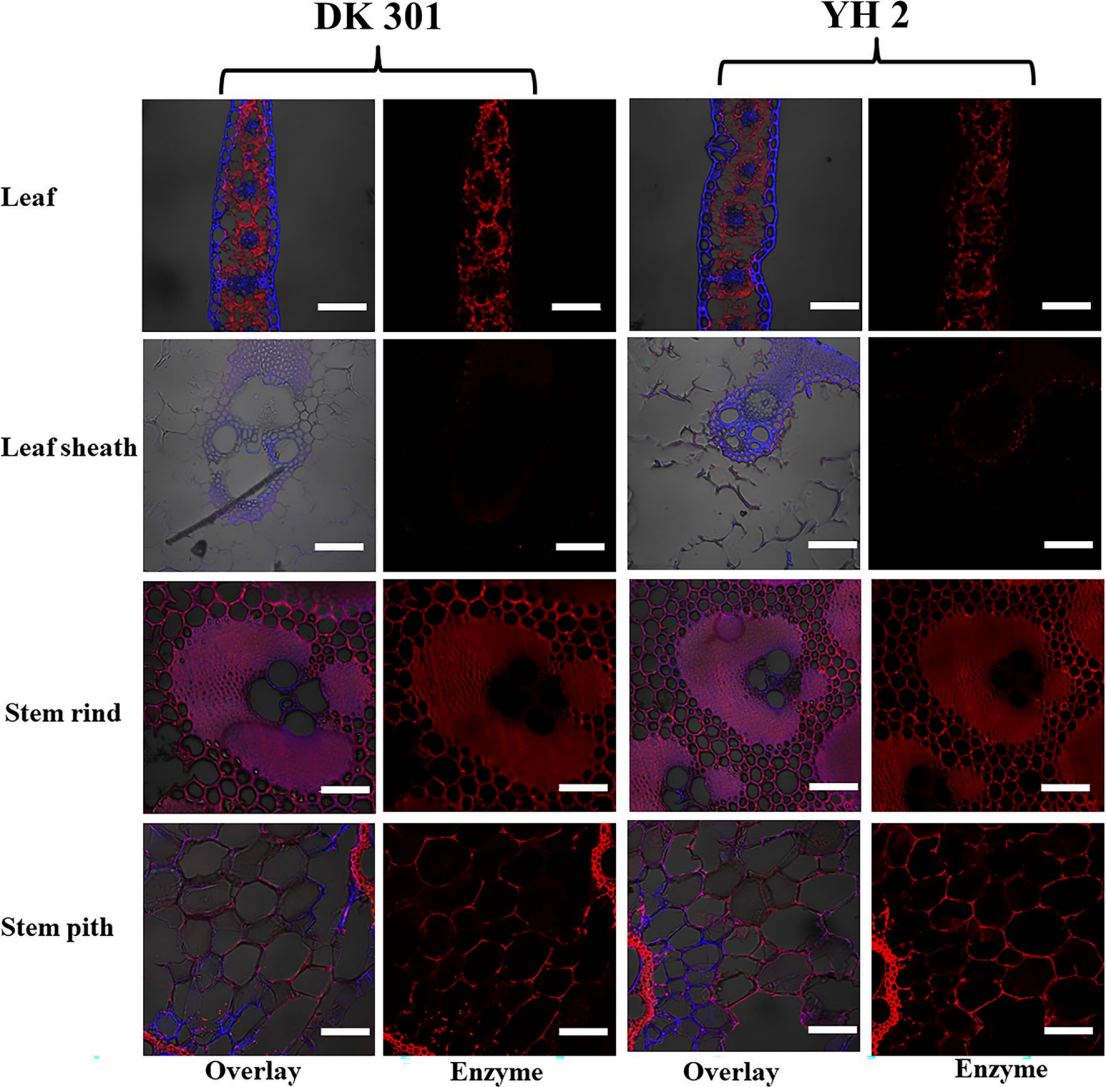
Cellulase adsorption on different fractions from the two corn cultivars photographed by CLSM. Scale bar = 100 μm

The labeled cellulase with the specific function of recognizing and adsorbing on cellulose was selected to observe the cellulase adsorption of different cell wall. The imaging results displayed that all the fractions of CS gave the complete structure after sectioning, except for the leaf sheath (Fig. 5). The transverse view exhibited the typical tissue structure of monocotyledons consisting of parenchyma cells and scattered vascular bundles. Parenchyma cells accounted for the half of stem rind and stem pith roughly. Additionally, leaf constituted of the mesophyll, vascular bundles and epidermal cells. Chemical mapping of chromophores on both the surface and inner fractions of cell wall was revealed by CLSM. The red fluorescence emission of DyLight-labeled enzyme was not affected by blue lignin autofluorescence. The variations of red fluorescence from the same fraction of DK or YH were similar, yet significant different within different fractions of the same cultivar. Precisely, the labeled cellulase interacted with the surface at or within cell wall of stem pith strongly, yet very weakly in the leaf sheath. The labeled cellulase was bound to the mesophyll and vascular bundles distinctly but not to the adjacent epidermal cells in leaf. Epidermal cells with special structure encrusted with waxes and cutin mainly envelop leaves, stem rind and leaf sheath. The cuticle is overlaid by wax deposits that play a role in resistance to microbes and insects, and water retention in the nature [26]. Thus, dermal cells provided rigidity, which was significantly resistant to enzyme adsorption.

From the view of plant anatomy, vascular bundles surrounding sclerenchyma tissue often have thickened and lignified secondary walls, whereas parenchyma cells only have primary walls and commonly do not have lignified secondary walls. Interestingly, more cellulase was adsorbed on the highly lignified vascular bundles than the less lignified parenchyma in the stem pith. However, Ding reports that general digestibility is significantly negatively correlated with lignin content of cell wall [27]. This might be caused by the non-productive binding of lignin in vascular bundles. Donaldson shows a moderate correlation between the enzyme adsorption and the cell wall histochemistry [28]. Our previous results also demonstrate that the lignin distribution, and interactions between lignin and cellulose could reduce the digestibility of cellulose [29]. These results revealed that the complicated physical–chemical properties of cell wall interface and its inter-relationship significantly affected the rankings of cellulase adsorption and also correlated to recalcitrance. The recalcitrance of sugar cane becomes weak gradually from the outer to the inner fractions [30]. Recalcitrance of cell wall in *Miscanthus* correlates to the tissue-specific distribution of lignin and development stage [31]. The heterogeneity of plant biomass highly depended on their origin, organ type, tissue trait, cell wall architecture, monomer composition, chemical bonds, its stage of development, etc., which further caused heterogeneity of the recalcitrance. Multiple architectures of cell wall, especially for the interface made the glycosyl hydrolase more difficult attack the plant cell wall. To understand the recalcitrance at cell wall interface might be the vital step for the selections of pretreatment conditions and enzymatic hydrolysis strategies.

## Conclusions

The compiled data revealed that the heterogeneity in structure and physicochemical properties of cell wall were distinct among organs or tissues of CS, and significantly correlated to the recalcitrance. Critical factors of cell wall had obvious impacts on cellulase adsorption that furthermore determined enzymatic digestion. Among a set of variables, pore size was shown as the predominant factor that affected the access of cellulase to polysaccharides within the cell wall. Thus, a holistic view of microscopic interface of cell wall was proposed, considering that concrete variable have interactive impacts on cellulase adsorption depending on overall interface structure assembly. Furthermore, a combination of many variables might contribute to saccharification efficiency, indicating that biorefineries should be the optimal choice to better exploit heterogeneity and optimize biomass flows.

## Methods

### Materials

Twelve corn (*Zea mays* L.) cultivars with different macroscopic phenotypes including dry weight, height and diameter were grown in experimental field of Helin, Inner Mongolia, China (Additional file 2: Table S1). The total aboveground biomass was harvested for this experiment when it was mature. The fresh corn straw without internode was selectively separated into four anatomical fraction including leaf, leaf sheath, stem rind and stem pith by hand, based on their macroscopic appearance and function (Additional file 1: Figure S1). After that the sample was washed by tap water and air dried. The tested samples were random mixed for sampling uniformity and further divided into two fractions for different analysis requirement. One fraction was ground to powder with size ranging from 0.84 to 0.42 mm through high-speed rotary cutting mill (BJ-300A, Baijie, China). The other fraction was sectioned and used for microscopy. Based on the results of saccharification ratio, the DK and YH were selected for the further physical–chemical analysis of cell wall. Cellulase (*Trichoderma viride* G) was bought from Shanghai Yuanye Co., Ltd. The activity of cellulase presenting a filter paper unit of 110.2 U/g was obtained following the classic procedure described by Ghose [32]. Cellulases (*Trichoderma reesei* ATCC 26,921) bought from Sigma-Aldrich and labeled DyLight 633™ provided by Thermo Fisher were used for cell wall staining. Direct Blue 1 (Pontamine Fast Sky Blue 6BX) and Direct Orange 15 (Pontamine Fast Orange 6RN) dyes were obtained from Pylam Products Co. Inc. (Garden City, NY). All other reagents were analytic grade and obtained from Shengkang Biotechnology Company (Huhhot, China) unless otherwise noted.

### Assessment of enzymatic digestibility

Enzymatic digestibility was determined via cellulase-mediated hydrolysis and quantitation of reducing sugars generated from cellulose and hemicelluloses. The enzymatic hydrolysis was performed in 50 mM sodium citrate (pH 4.8) with solid consistency of 5% (w/w), enzyme loading of 20 FPU/g and at 50 ^○^C for 48 h. The reducing sugars were assayed according to the 3,5-dinitrosalicylic acid method described by Miller [33, 34]:

1 $$ {\text{Saccharification}}\,{\text{ratio}}\,(\% ) = \left( {m_{{\text{g}}} \times 100} \right)/\left( {m_{{\text{H - Cel}}} + m_{{{\text{Cel}}}} } \right), $$

where *m*_g_ represented amount (g) of the generated reducing sugars during enzymatic hydrolysis, and *m*_H-Cel_ and *m*_Cel_ represented weight of hemicelluloses and cellulose in the tested sample (g), respectively.

### Chemical composition analysis

Chemical characterization analysis was performed according to a protocol recommended by Van Soest [35]. Various dry samples were treated by neutral buffered detergent solution, hydrochloric acid (2 M) and sulfuric acid (7.34 M) to determine neutral detergent fiber (NDF), acid detergent fiber (ADF), and acid detergent lignin (ADL) in sequence. The ash content was assayed by heating the ADL at 550 °C for 180 min. The contents of neutral detergent soluble (NDS), hemicelluloses, cellulose, and lignin were calculated by subtracting the corresponding values from the initial dry weight, NDF, ADF, ADL and ash fractions in sequence.

### Crystallinity analysis

Powder X-ray diffractometer (X’Pert PRO, PANalytical, Netherlands) was used to measure the Segal crystallinity index (CrI) and crystallite dimension (CD). Scans were performed in triplicate at 1°/min from 5° to 40° 2*θ* with a step size of 0.02°. The CrI was calculated from the XRD patterns based on the empirical peak-height method (Eq. 2) [36]. The dimensions of the crystallites in various samples were determined through Scherrer method (Eq. 3):

2 $$ CrI = \left( {I_{{{\text{total}}}} - I_{{{\text{amor}}}} } \right)/I_{{{\text{total}}}} . $$

*I*_total_ was the diffraction intensity of major peak at 2*θ* between 22° and 23° for cellulose Iβ. *I*_amor_ was the diffraction intensity of the minima between the major peak and secondary peaks.

3 $$ \tau = \left( {K \times \lambda } \right)/\left( {\beta \times \cos \theta } \right), $$

where *K* was a constant value of 0.94; *λ* was the X-ray wavelength of 0.1542 nm; *β* was peak width of diffraction band at the half maximum height; *τ* represented the crystallite dimension; and *θ* was the Bragg angle fitted from the major peak by Inc Jade 6.5 software.

### Degree of polymerization determination

Dry powders were extracted by 4 M KOH solution containing sodium borohydride (1.0 mg/mL) at 25 °C for 1 h, with solid-to-liquid ratio of 1:10. After being extracted for repeated twice, the alkaline slurry was further washed by distilled water and extracted with 10 mL 8% NaClO_2_ solution (containing 1.5% glacial acetic acid) at 25 °C for 1 h to obtain crude cellulose. The degree of polymerization of the crude cellulose was determined by an Ubbelohde viscosity meter with the modified viscosity method at ambient temperature (around 25 °C) [37].

### Simons’ stain

The overall porosity was measured by Simons’ stain [38]. The DB and DO dyes using the same amount of 10 mg/ml were mixed with the ratio of 1:1. Precisely 0.1 g of dry powders and increasing volumes of the mixed dye solutions (0.25, 0.5, 0.75, 1.00, 1.50 ml) were added into the centrifuge tubes. Phosphate buffered saline solution (pH 6, 0.2 M PO_4_, 1.40 M NaCl) was added into each tube to make the final volume up to 10 mL. After incubating at 70 °C for 6 h with gentle shaking, the tubes were centrifuged at 6200×*g* for 10 min. The absorbance of the supernatant solution in the tube was measured at the maximum wave length of 455 nm and 624 nm for DO and DB, respectively. The dye adsorption isotherm was determined to calculate the maximum DB and DY dyes.

### Determination of porous parameters of corn straw

The porous parameters of various samples were evaluated by mercury porosimeter (AutoPore IV 9500, Micromeritics Instrument Corporation, USA), with a contact angle of 130° and pressure ranging 1 × 10^–1^ to 6.1 × 10^4^ psia. Intrusion pressure was directly converted to the relevant pore size through the Washburn equation [39].

### Sectioning and cellulase labeling

To visualize the enzyme adsorption, pieces (around 5 mm) were cut from the middle portion of each fresh sample and embedded by tissue freezing medium (Leica, 4 fl) under – 20 °C. The transverse slices (8 μm in thickness) of different samples were prepared using Leica cryostat (Leica, RM2015) for confocal laser scanning microscopy (CLSM, LSM-710, ZEISS, Germany). The cellulase labeling procedure was carried out as the manufacturer’s instructions adopted by Donaldson with a slight modification [28]. Concretely, the freeze-dried cellulase powder was dissolved in borate-phosphate buffered saline (0.05 M, pH 7.5) and mixed with DyLight 633™ in the tube covered with aluminum foil at ambient temperature (around 25 °C) for 60 min, with gentle shaking [40]. And then the mixture was passed through the supplied resin and centrifuged multiple times (6200×*g*, 15 min) to separate the unlabeled fluorophore. The purified labeled cellulase was kept at 4 °C avoiding light. Cellulase (the given average molecular weight of 65 kDa), with fluorophore-to-protein molar ratio of 7.48:1, was used to label the section of samples for 60 min. The distribution of the label cellulase on cell wall was described by CLSM, using the identical instrument settings: 10 × /1.40 NA Plan-Apochromatic objective lens, pinhole size of 1 AU, 405 nm and 633 nm sequential excitation, and 410–480 nm (blue lignin autofluorescence), and 650–750 (red DyLight 633™ labeled cellulase fluorescence) emission. Lignin fluorescence and labeled enzyme were visualized by sequential excitation, without any significant bleed-through of signals. All images were shown under the uniform condition with optimal intensity.

### Statistical analysis

Heat map and dendrogram were plotted using the original “heatmap” function shipped with R installation, based on the hierarchical clustering results of saccharification ratio. Biological triplicates were selected for each corn cultivar. Chemical and physical analysis was carried out in technical triplicates to ensure reproducibility. The data were expressed as the mean of triplicates ± standard deviation (SD). Statistical analysis was carried out using the SPSS statistical software with one-way ANOVA. The results were considered statistically significant, when *P* value of the differences was lower than 5% at the 95% confidence interval.

## Supplementary Information

**Additional file 1: Figure S1.** Selective structure fractionation of corn straw.

**Additional file 2: Table S1.** Macroscopic phenotypes of twelve corn (*Zea mays* L.) cultivars.

**Additional file 3: Table S2.** Saccharification ratios for four anatomical fractions of DK 301 and YH 2.

## Data Availability

All data generated or analyzed during this study are included in this published article and its Additional files.

## Abbreviations

NDS: Neutral detergent soluble
H-Cel: Hemicelluloses
Cel: Cellulose
Lig: Lignin
CrI: Crystallinity index
DP: Degree of polymerization
CD: Crystallite dimension
DB: Direct Blue 1
DO: Direct Orange 14
SD: Standard deviation
CLSM: Confocal laser scanning microscopy
FPU: Filter paper unit

## References

[1] Arpia AA, Chen WH, Lame SS, Rousset P, de Lunah MDG (2021). Sustainable biofuel and bioenergy production from biomass waste residues using microwave-assisted heating: a comprehensive review. Chem Eng J.

[2] Dahmen N, Lewandowski I, Zibek S, Weidtmann A (2019). Integrated lignocellulosic value chains in a growing bioeconomy: status quo and perspectives. GCB Bioenergy.

[3] Qin S, Giri BS, Patel AK, Sar T, Liu H, Chen H (2020). Resource recovery and biorefinery potential of apple orchard waste in the circular bioeconomy. Bioresource Technol.

[4] Banu JR, Kavitha PS, Tyagi VK, Gunasekaran M, Karthikeyan OP (2021). Lignocellulosic biomass based biorefinery: a successful platform towards circular bioeconomy. Fuel.

[5] Himmel ME, Ding SY, Johnson DK, Adney WS, Nimlos MR, Brady JW (2007). Biomass recalcitrance: engineering plants and enzymes for biofuels production. Science.

[6] Jin K, Kong L, Liu X, Jiang Z, Tian G, Yang S (2019). Understanding the xylan content for enhanced enzymatic hydrolysis of individual bamboo fiber and parenchyma cells. ACS Sustain Chem Eng.

[7] Crowe JD, Feringa N, Pattathil S, Merritt B, Foster C, Dines D (2017). Identification of developmental stage and anatomical fraction contributions to cell wall recalcitrance in switchgrass. Biotechnol Biofuels.

[8] Olatunji KO, Ahmed NA, Ogunkunle O (2021). Optimization of biogas yield from lignocellulosic materials with different pretreatment methods: a review. Biotechnol Biofuels.

[9] Tingley JP, Low KE, Xing X, Abbott DW (2021). Combined whole cell wall analysis and streamlined in silico carbohydrate-active enzyme discovery to improve biocatalytic conversion of agricultural crop residues. Biotechnol Biofuels.

[10] Devaux MF, Jamme F, André W, Bouchet B, Alvarado C, Durand S (2018). Synchrotron time-lapse imaging of lignocellulosic biomass hydrolysis: tracking enzyme localization by protein autofluorescence and biochemical modification of cell walls by microfluidic infrared microspectroscopy. Front Plant Sci.

[11] Gao Y, Lipton AS, Wittmer Y, Murray DT, Mortimer JC (2020). A grass-specific cellulose–xylan interaction dominates in sorghum secondary cell walls. Nat Commun.

[12] Chahal A, Ciolkosz D, Puri V, Jacobson M, Liu J (2021). Mechanical characteristics of wood-bark interface of shrub willow. Ind Crop Prod.

[13] Li T, Liu N, Ou X, Zhao X, Qi F, Huang J (2018). Visualizing cellulase adsorption and quantitatively determining cellulose accessibility with an updated fungal cellulose-binding module-based fluorescent probe protein. Biotechnol Biofuels.

[14] Ruthes AC, Rudjito RC, Rencoret J, Gutiérrez A, del Río JC, Jiménez-Quero A (2020). Comparative recalcitrance and extractability of cell wall polysaccharides from cereal (wheat, rye, and barley). ACS Sustain Chem Eng.

[15] Li D, Long L, Ding S (2020). Alkaline organosolv pretreatment of different sorghum stem parts for enhancing the total reducing sugar yields and p-coumaric acid release. Biotechnol Biofuels.

[16] Hodgson-Kratky K, Papa G, Rodriguez A, Stavila V, Simmons B, Botha F (2019). Relationship between sugarcane culm and leaf biomass composition and saccharification efficiency. Biotechnol Biofuels.

[17] Li Y, Merrettig-Bruns U, Strauch S, Kabascib S, Chen H (2015). Optimization of ammonia pretreatment of wheat straw for biogas production. J Chem Technol Biotechnol.

[18] Shah R, Bhagia S, Keum JK, Pingali SV, Ragauskas AJ, Davison BH (2020). Structural insights into low and high recalcitrance natural poplar variants using neutron and X-ray scattering. ACS Sustain Chem Eng.

[19] Silva LA, Gasparini K, Assis C, Ramos R, Kist V, Pereria-Barbosa MH (2017). Selection strategy for indication of crosses between potential sugarcane genotypes aiming at the production of bioenergy. Ind Crop Prod.

[20] Chen X, Xiong L, Li H, Zhang L, Yuan G, Chen X (2020). The inhibitory effect of xylan on enzymatic hydrolysis of cellulose is dependent on cellulose ultrastructure. Cellulose.

[21] Chandra R, Ewanick S, Hsieh C, Saddler JN (2008). The characterization of pretreated lignocellulosic substrates prior to enzymatic hydrolysis, part 1: a modified Simons’ staining technique. Biotechnol Prog.

[22] Arantes V, Saddler JN (2011). Cellulose accessibility limits the effectiveness of minimum cellulase loading on the efficient hydrolysis of pretreated lignocellulosic substrates. Biotechnol Biofuels.

[23] Arantes V, Saddler JN (2010). Access to cellulose limits the efficiency of enzymatic hydrolysis: the role of amorphogenesis. Biotechnol Biofuels.

[24] Sun D, Alam A, Tu Y, Zhou S, Wang Y, Xia T (2017). Steam-exploded biomass saccharification is predominately affected by lignocellulose porosity and largely enhanced by tween-80 in *miscanthus*. Bioresour Technol.

[25] Zhao JY, Chen HZ (2013). Correlation of porous structure, mass transfer and enzymatic hydrolysis of steam exploded corn stover. Chem Eng Sci.

[26] Wilson LA, Deligey F, Wang T, Cosgrove DJ (2021). Saccharide analysis of onion outer epidermal walls. Biotechnol Biofuels.

[27] Ding SY, Liu YS, Zeng Y, Himmel ME, Baker JO, Bayer EA (2012). How does plant cell wall nanoscale architecture correlate with enzymatic digestibility?. Science.

[28] Donaldson L, Vaidya A (2017). Visualising recalcitrance by colocalisation of cellulase, lignin and cellulose in pretreated pine biomass using fluorescence microscopy. Sci Rep.

[29] Li G, Zhang Y, Zhao C, Xue H, Yuan L (2020). Chemical variation in cell wall of sugar beet pulp caused by aqueous ammonia pretreatment influence enzymatic digestibility of cellulose. Ind Crop Prod.

[30] Costa THF, Masarin F, Bonifácio TO, Milagres AMF, Ferraz A (2013). The enzymatic recalcitrance of internodes of sugar cane hybrids with contrasting lignin contents. J Clean Prod.

[31] da Costa RMF, Pattathil S, Avci U, Lee SJ, Hazen SP, Winters A (2017). A cell wall reference profile for *miscanthus* bioenergy crops highlights compositional and structural variations associated with development and organ origin. New Phytol.

[32] Ghose TK (1987). Measurement of cellulase activities. Pure Appl Chem.

[33] Miller GL (1959). Use of dinitrosalicylic acid reagent for determination of reducing sugar. Anal Biochem.

[34] Xu C, Ma F, Zhang X, Chen S (2010). Biological pretreatment of corn stover by *Irpex lacteus* for enzymatic hydrolysis. J Agric Food Chem.

[35] Van Soest PJ, Wine RH (1967). Use of detergents in the analysis of fibrous feeds. IV. Determination of plant cell-wall constituents. J Assoc Off Anal Chem.

[36] Segal L, Creely JJ, Martin AE, Conrad CM (1959). An empirical method for estimating the degree of crystallinity of native cellulose using the X-ray diffractometer. Text Res J.

[37] Wang W, Chen X, Donohoe BS, Ciesielski PN, Katahira R, Kuhn EM (2014). Effect of mechanical disruption on the effectiveness of three reactors used for dilute acid pretreatment of corn stover part 1: chemical and physical substrate analysis. Biotechnol Biofuels.

[38] Meng X, Foston M, Leisen J, Demartini J, Wyman CE, Ragauskas AJ (2013). Determination of porosity of lignocellulosic biomass before and after pretreatment by using Simons’ stain and NMR techniques. Bioresour Technol.

[39] Meng X, Wells JT, Sun Q, Huang F, Ragauskas A (2015). Insights into the effect of dilute acid, hot water and alkaline pretreatment on cellulose accessible surface area and overall porosity of *Populus*. Green Chem.

[40] Li GH, Sun YX, Guo WJ, Yuan L (2018). Comparison of various pretreatment strategies and their effect on chemistry and structure of sugar beet pulp. J Clean Prod.

