# Supplementary figures and images for: Physical–chemical properties of cell wall interface significantly correlated to the complex recalcitrance of corn straw
Source: Biotechnol Biofuels. 2021 Oct 1;14:196. doi: 10.1186/s13068-021-02047-0 (PMC8487139; doi:10.1186/s13068-021-02047-0)

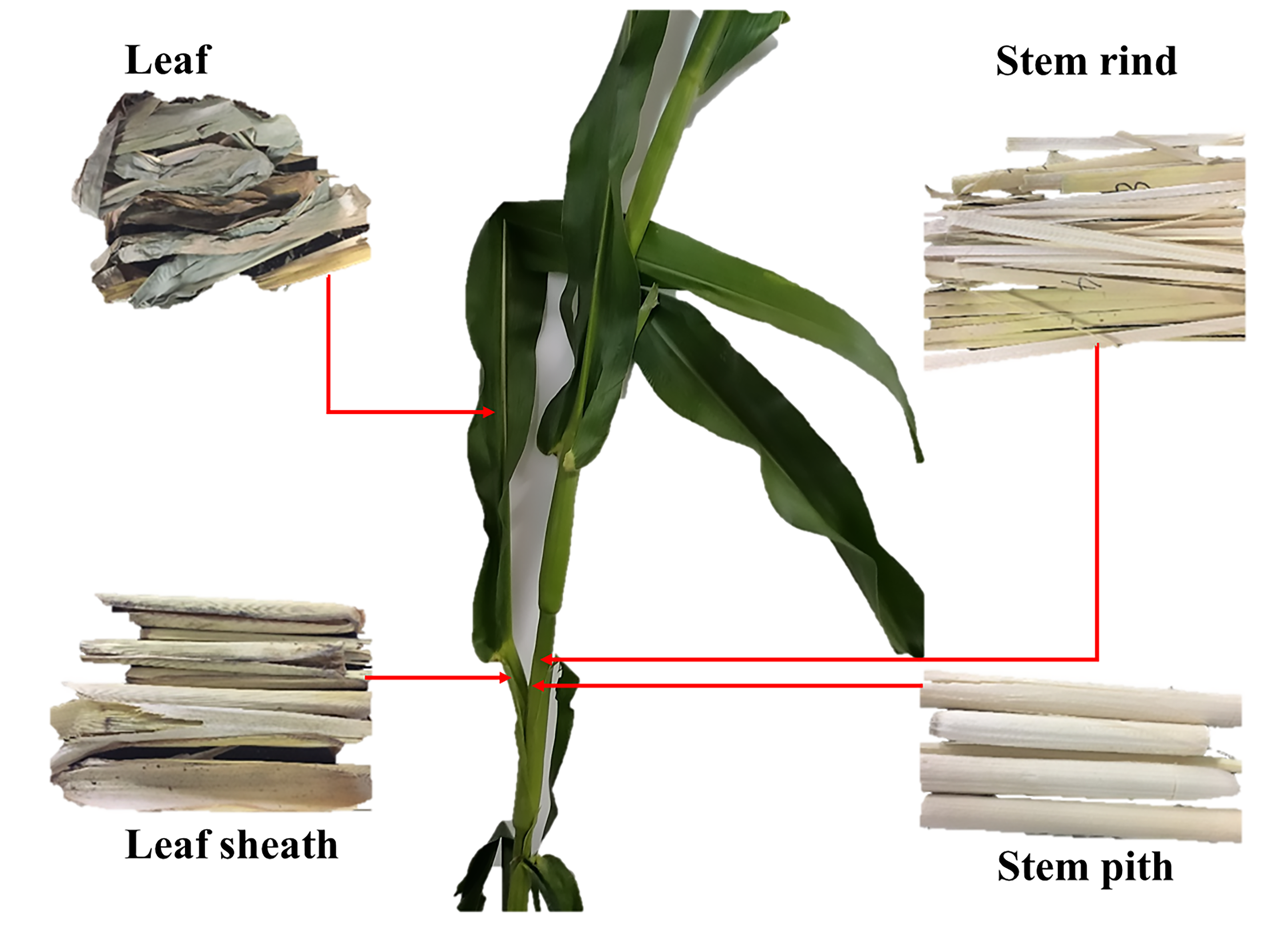

Supplement: Supplementary file 1 — Additional file 1: Figure S1. Selective structure fractionation of corn straw. [file 13068_2021_2047_MOESM1_ESM.tif]
