# Supplementary material for: Physical–chemical properties of cell wall interface significantly correlated to the complex recalcitrance of corn straw
Source: Biotechnol Biofuels. 2021 Oct 1;14:196. doi: 10.1186/s13068-021-02047-0 (PMC8487139; doi:10.1186/s13068-021-02047-0)
Supplement: Supplementary file 2 — Additional file 2: Table S1. Macroscopic phenotypes of twelve corn (Zea mays L.) cultivars. [file 13068_2021_2047_MOESM2_ESM.docx]

**Additional file 2: Table S1. Macroscopic phenotypes of twelve corn (*Zea mays* L.) cultivars**

| **Cultivar** | **Dry Weight (g)** | **Height (cm)** | **Diameter (mm)** |
| --- | --- | --- | --- |
| CF 22 | 150.15±16.82 | 354.48±0.71 | 23.52±0.68 |
| DK 301 | 110.24±5.21 | 270.52±17.68 | 23.93±1.52 |
| FY 968 | 139.08±11.48 | 332.52±17.68 | 20.72±2.25 |
| JA 588 | 143.92±32.88 | 342.50±20.51 | 21.8±0.028 |
| JL 327 | 102.56±23.89 | 256.51±4.95 | 16.28±2.16 |
| KXA 4574 | 111.35±1.63 | 271.54±30.41 | 21.21±1.61 |
| LH 1 | 141.79±6.07 | 335.49±6.36 | 22.38±2.99 |
| SN 211 | 140.99±21.43 | 320.53±10.61 | 22.29±1.57 |
| SY 801 | 122.58±7.65 | 278.00±21.21 | 18.07±2.04 |
| XY 335 | 137.77±7.38 | 323.75±3.89 | 21.35±0.53 |
| YH 2 | 151.90±15.06 | 351.00±9.90 | 22.29±0.014 |
| ZX 618 | 124.94±1.98 | 298.50±4.95 | 16.45±2.33 |
