# Supplementary material for: Physical–chemical properties of cell wall interface significantly correlated to the complex recalcitrance of corn straw
Source: Biotechnol Biofuels. 2021 Oct 1;14:196. doi: 10.1186/s13068-021-02047-0 (PMC8487139; doi:10.1186/s13068-021-02047-0)
Supplement: Supplementary file 3 — Additional file 3: Table S2. Saccharification ratios for four anatomical fractions of DK 301 and YH 2. [file 13068_2021_2047_MOESM3_ESM.docx]

**Additional file 3: Table S2. Saccharification ratios for** four anatomical fractions **of DK 301 and YH 2**

| **Cultivar** | **Fraction** | **Saccharification ratio (%)** |
| --- | --- | --- |
| DK 301 | Leaf | 33.22±0.60^a^ |
|  | Leaf sheath | 19.30±0.40^b^ |
|  | Stem rind | 37.47±1.26^c^ |
|  | Stem pith | 38.80±0.15^c^ |
| YH 2 | Leaf | 23.32±0. 32^d^ |
|  | Leaf sheath | 19.92±0. 12^b^ |
|  | Stem rind | 16.09±0.19^e^ |
|  | Stem pith | 25.97±0.80^f^ |

The data labeled by the different superscripts (a–g) were different from each other (*p*<0.05).
